# Nanoscale Room-Temperature Na Dynamics in Layered Ruthenates Na_1_RuO_3_ and Na_1.5_RuO_3_

**DOI:** 10.3390/nano16100577

**Published:** 2026-05-08

**Authors:** Mohammad Hussein Naseef Assadi

**Affiliations:** 1 RIKEN Center for Emergent Matter Science (CEMS), 2–1 Hirosawa, Wako 351-0198, Saitama, Japan; h.assadi.2008@ieee.org; 2 Chemistry Department, Faculty of Engineering and Natural Sciences, Istinye University, Sariyer 34396, Istanbul, Türkiye

**Keywords:** AIMD, density functional theory, molecular dynamics, layered materials, Na diffusion, metaGGA

## Abstract

Understanding the atomic-scale ionic motion and transport in layered transition-metal oxides is essential for elucidating structural stability and electronic behaviour in complex systems. Here, we investigate nanoscale Na dynamics in Na_1_RuO_3_ and Na_1.5_RuO_3_ using room-temperature ab initiomolecular dynamics at the r^2^SCAN + *U* level. While Na mobility plays a key role in local coordination, its nanoscale mechanism remains nuanced and unexplored. Our simulations show that Na ions undergo pervasive rattling, with Na_1.5_RuO_3_ enabling exploration of larger volumes and exhibiting incipient migration compared to the more confined behaviour in Na_1_RuO_3_. In addition, oxygen’s contribution to redox capacity decreases from 43% to 24% with increasing Na content. These nanoscale insights demonstrate that tuning the local ionic environment governs charge compensation and dynamical response in ruthenate frameworks, with direct implications for the design of Na-ion battery cathodes.

## 1. Introduction

Understanding the Na^+^ dynamics in layered sodium oxides is essential for advancing sodium-ion batteries because Na offers a low-cost, earth-abundant alternative to Li, yet its larger ionic radius and lower ionisation potential impose structural and electrochemical challenges that must be overcome to achieve a competitive energy density [1,2,3]. In 4d transition-metal oxides such as Na_x_RuO_3_, strong 4d-2p hybridisation in the Ru–O bond lifts un-hybridised O 2p states toward the Fermi level, enabling reversible oxygen redox that can supply a large portion, or even the majority, of the charge compensation and deliver capacities far beyond those achievable with cationic redox alone [4]. Na excess, in nearly fully sodiated compound, further enhances this effect: when more than three Na ions coordinate O, the O 2p band broadens and contributes nearly twice as much as Ru 4*d* states to charge compensation, raising the average voltage (2.458 V for the disordered hexagonal phase versus 2.243 V for the ordered monoclinic phase) [5,6]. The concurrent O + Ru redox observed in Na_x_RuO_3_, demonstrates that Na dynamics and local Na–O coordination directly control the electrochemical window [7,8].

Insights from the widely studied Na_x_CoO_2_ system underline the broader relevance of Na dynamics. For instance, NMR and diffraction studies reveal that Na layers in Na_0.8_CoO_2_ undergo a first-order “melting” transition near 291 K, where fast two-dimensional (2-D) Na diffusion homogenises the local electric-field environment, profoundly affecting magnetic and electronic properties [9]. In Na_0.7_CoO_2_, two structural transitions (∼290 K and ∼400 K) switch Na diffusion from quasi-one-dimensional pathways to fully 2-D motion, while the CoO_2_ slabs remain essentially unchanged, highlighting the decoupling of Na-layer dynamics from the transition-metal framework [4,5,6]. Temperature-driven Na-site ordering also drives anomalous phenomena such as negative thermal expansion and significant off-centre shifts of Na(2) ions in Na_0.75_CoO_2_, linking subtle Na-sublattice rearrangements to lattice strain and electronic response [10].

Similar to the case of Na_x_CoO_2_, Na-driven structural evolutions have direct implications for the design of Na_x_RuO_3_ based cathode materials. For instance, the honeycomb-ordered O-Na2RuO_3_ phase delivers 1.3 *e*^−^ (∼180 $mA\;h\;g^{-1}$) because ordered Na-rich slabs stabilise an intermediate ilmenite Na_1_RuO_3_, shorten O–O distances and raise the O–O antibonding orbital to the Fermi level, thereby triggering stable oxygen redox by preventing O ions from bonding to each other [11]. By contrast, disorder induces “strain frustration” that suppresses this mechanism and reduces capacity [12]. The Na-excess and covalency strategy identified in Ru-oxides can therefore be generalised: engineering Na coordination environments (through controlled Na loading, cation ordering, or phase selection) offers a route to activate oxygen redox while avoiding detrimental O_2_ evolution, as predicted in ilmenite Na_1_NbO_3_ and Na_1_VO_3_, which exhibit high voltages (up to 5.9 V) and robust cyclability [6,13].

Overall, Na dynamics govern both the structural stability and the redox chemistry of layered sodium oxides. Lessons from Na_x_CoO_2_, where Na mobility dictates phase transitions, magnetic ordering, and electronic structure, highlight that precise control of Na content, ordering, and diffusion pathways is a prerequisite for harnessing the full capacity of Na_x_RuO_3_ and related high-performance sodium-ion battery cathodes. Motivated by this perspective, we employ long-timescale, room-temperature ab initio molecular dynamics simulations to directly elucidate sodium transport and its coupling to the local structural and electronic environment in Na_1_RuO_3_ and Na_1.5_RuO_3_.

## 2. Materials and Methods

Spin-polarised ab initio molecular dynamics (AIMD) simulations were performed using the VASP package [14,15]. After an initial equilibration period, production runs of 15 ps were carried out for both Na_1_RuO_3_ and Na_1.5_RuO_3_ supercells, using a time step of 0.5 fs, corresponding to 30,000 MD frames. The electronic self-consistency convergence criterion was set to $10^{-6}$ eV per supercell. All simulations were conducted at 300 K in the canonical ensemble [16], with an Andersen thermostat for temperature control [17]. The simulation runs consumed ∼600,000 CPU hours. The energy and trajectory output files were analysed using the Atomic Simulation Environment (ASE) library [18].

Exchange-correlation was treated at the meta-GGA level using the r^2^SCAN functional [19]. On-site electronic correlations of the Ru 4d states were accounted for through an ad hoc Hubbard correction employing the Liechtenstein–Anisimov–Zaanen [20] scheme, with U=3 eV and J=1 eV. These *U* and *J* values were chosen to reproduce the measured voltages of the hexagonal Na_x_RuO_3_ [6]. The electronic density of states (DOS) was obtained by averaging the DOS over all frames after equilibration. The tetrahedron method with Blöchl corrections without smearing was employed for DOS calculation to ensure accuracy. The suitability of the chosen *U* parameters and the AIMD setup has been validated in our previous work [13,21]. To aid with convergence, the electronic minimisation was performed using a simultaneous all-band Davidson diagonalisation scheme, in which all Kohn–Sham orbitals are updated concurrently. Moreover, non-spherical contributions from the gradient corrections inside the PAW spheres were also included. Similar first-principles approaches have been successfully applied in other layered systems [22].

## 3. Results and Discussion

**Figure 1:**
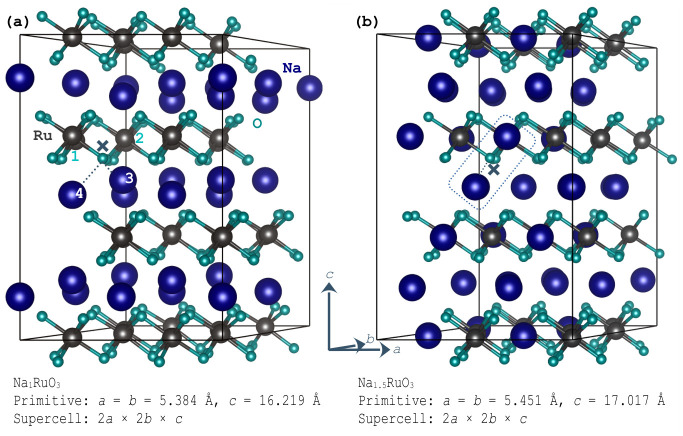
Optimised supercells used to initiate the molecular dynamics runs. (**a**) Na_1_RuO_3_ and (**b**) Na_1.5_RuO_3_. The optimised lattice parameters of the primitive cells, along with the supercell dimensions, are provided. In (**a**), one oxygen ion is marked with a cross, and its coordinating Ru^4+^ and Na^+^ cations are numbered. In (**b**), an oxygen atom with Na–O–Na coordination environment is marked with a dashed enclosure.

Na_1_RuO_3_ and Na_1.5_RuO_3_ are hexagonal ruthenate phases that emerge during the sodiation and desodiation of Na_x_RuO_3_ cathodes, with x=0.5 and 2 representing the compositional extremes. Na_1_RuO_3_, shown in Figure 1a, adopts an ilmenite structure and crystallises in the $R\bar{3}$ space group, in which only two-thirds of the octahedral cation sites are occupied [6]. As a result, every oxygen atom is under-coordinated, with two Ru^4+^ and two Na^+^ neighbours. This “cation-vacancy” topology lifts non-bonding O 2p states toward the Fermi level, such that oxygen accounts for approximately two-thirds of the charge compensation during desodiation [13].

By contrast, Na-excess layered phases such as Na_1.5_RuO_3_ (Figure 1b) adopt a sodium-deficient O3-type $R\bar{3}m$ slab, in which excess Na occupies one third of the transition-metal sites [4]. In this case, half oxygen atoms are coordinated by more than three Na^+^ ions, and this unusual Na–O–Na environment similarly raises the O 2p band, making oxygen the dominant redox species [23]. Structurally, both compounds share edge-sharing RuO_6_ frameworks and strong Ru 4d-O 2p covalency, which stabilise oxidised oxygen against the detrimental release of O_2_ during the redox cycle.

Despite these similarities, the two compounds differ in Na content, vacancy ordering (intrinsic octahedral vacancies in the ilmenite phase versus long-range honeycomb ordering in Na-excess phases), and the resulting lattice evolution during the sodiation/desodiation cycle. Nonetheless, both materials exploit a similar electronic principle, i.e., rendering O 2p states redox-labile, either through oxygen undercoordination or a Na surplus. The AIMD simulations presented here aim to further elucidate how differences in Na content and lattice-site occupancy govern sodium dynamics at room temperature and, consequently, influence the redox mechanism.

**Figure 2:**
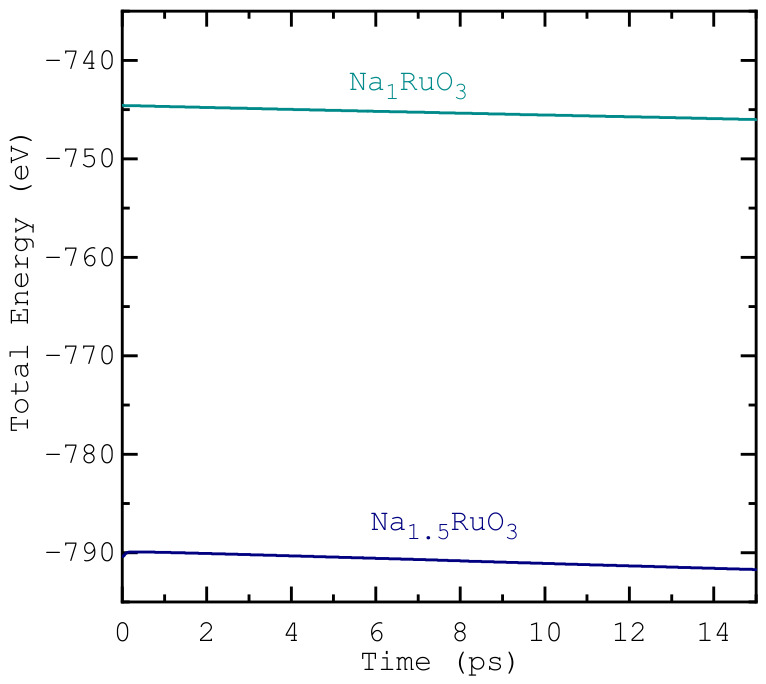
Energy vs. time throughout the simulation runs. The energy plateaus indicate convergence.

**Figure 3:**
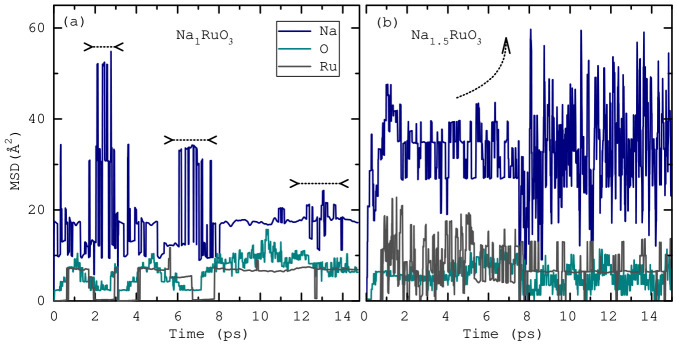
Mean squared displacements (MSD) for (**a**) Na_1_RuO_3_ and (**b**) Na_1.5_RuO_3_ throughtout the simulation runs. The horizontal bars in (**a**) indicate MSD plateaus associated with local rattling motions of Na ions, while the curved arrow in (**b**) highlights an increase in the Na vibrational amplitude, likely arising from migration into a larger local void within the non-close-packed structure.

In the equilibrated regime, shown in Figure 2, the total energy during the AIMD runs fluctuated by less than 1% relative to its mean, demonstrating numerical stability for both Na_1_RuO_3_ and Na_1.5_RuO_3_ runs, and ensuring that the sampled trajectories provide a reliable basis for analysing structural and dynamical properties. A useful metric in comparing the relative motion of atoms in a structure is the mean-squared displacement (MSD) values for each atom. MSD(*t*) is defined as $\langle {\left|r\left(t\right)-r\left(0\right)\right|}^{2}\rangle$ averaged over all atomic species in a supercell during the AIMD run. As shown in Figure 3, Na exhibits three-fold larger MSD values than the framework atoms, highlighting sodium’s enhanced mobility. In Na_1_RuO_3_, the time-averaged MSDs are 17.633 ${Å}^{2}$ for Na, 6.700 ${Å}^{2}$ for O, and 5.102 ${Å}^{2}$ for Ru, whereas in Na_1.5_RuO_3_ these values increase to 38.238, 11.032, and 12.716 ${Å}^{2}$, respectively. These results clearly demonstrate that Na ions are significantly more mobile than the Ru–O framework, with Na dynamics becoming increasingly pronounced in Na_1.5_RuO_3_.

Furthermore, the calculated Na MSD exhibits sub-diffusive regimes associated with vibrational and rattling motions, as evidenced by nearly flat MSD plateaus. In Na_1_RuO_3_ (Figure 3a), three plateaus with decreasing amplitudes are observed, as indicated by horizontal arrows. In Na_1.5_RuO_3_ (Figure 3b), two plateaus with increasing amplitudes are visible. The large Na rattling amplitudes originate from the non-close-packed coordination environment of Na ions and their largely non-directional Coulombic interactions with surrounding atoms, instead of directional covalent bonding. This tendency is reflected in the average Na–O coordination numbers of 2.35 for Na_1_RuO_3_ and 3.40 for Na_1.5_RuO_3_, calculated using a cutoff of 2.6 Å. Given the limited simulation time and finite supercell size inherent to AIMD, true long-range Na diffusion is not observed. Nonetheless, a clear contrast is evident between the bounded MSDs of Ru and O atoms and the pronounced rattling behaviour of Na ions.

**Figure 4:**
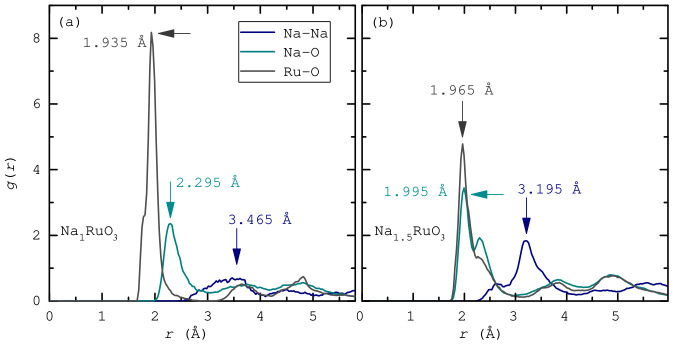
Calculated partial radial distribution functions g(r) for Na–Na, Na–O, and Ru–O, (**a**) Na_1_RuO_3_ and (**b**) Na_1.5_RuO_3_.

The radial distribution functions, shown in Figure 4, reveal some noticeable structural differences between Na_1_RuO_3_ and Na_1.5_RuO_3_. In Na_1.5_RuO_3_, the Na–Na correlations are more pronounced and slightly shifted to shorter distances (∼3.20 Å vs. ∼3.47 Å), indicating increased Na–Na interactions and a more crowded alkali substructure. The Na–O peak also becomes sharper and more defined, suggesting stronger or more ordered Na–O coordination. In contrast, the Ru–O bond distance remains relatively consistent in both systems (∼1.93–2.00 Å), implying that the RuO_6_ framework is largely preserved. Overall, the additional Na in Na_1.5_RuO_3_ primarily affects the Na environment, enhancing short-range ordering and interactions without significantly altering the Ru–O local structure. However, despite tighter packing and higher coordination with O, Na in Na_1.5_RuO_3_ enjoys a greater range of motion, as elucidated by the MSD analysis.

The self-van Hove correlation function $G_{s}\left(r,t\right)$, which can further help in understanding the Na^+^ ion dynamics, is the probability density that a particle originally at the origin will be found a distance *r* away after time *t* [24]. In other words, the van Hove correlation function describes the time-dependent probability of finding a particle at position *r* at time *t*, given that a particle was located at the origin at time t=0, thereby capturing how density fluctuations propagate through a many-body system in both space and time [25]. $G_{s}\left(r,t\right)$ is obtained from molecular-dynamics trajectories by histogramming displacements: $G_{s}\left(r,t\right)=N^{-1}\sum_{i}\langle \delta [r-|r_{i}\left(t\right)-r_{i}\left(0\right)|]\rangle$. For very short times $G_{s}\left(r,t\right)$ collapses to a Dirac delta, reproducing the radial distribution at t=0; at longer times it broadens from ballistic to diffusive Gaussian form, with mean-square displacement $\langle r^{2}\left(t\right)\rangle =6Dt$ yielding the self-diffusion coefficient, which is not applicable to our simulation due to restricted time scale. Obtaining a reliable diffusion coefficient typically requires simulations on nanosecond time scales, which are generally accessible only with classical molecular dynamics [26,27]. However, tracking the evolution of peak heights, widths, and shapes still reveals valuable insights such as cage-breaking, hopping, dynamic heterogeneity, and relaxation times. In its essence, $G_{s}\left(r,t\right)$ converts atomic motions into interpretable transport metrics for each atomic species.

**Figure 5:**
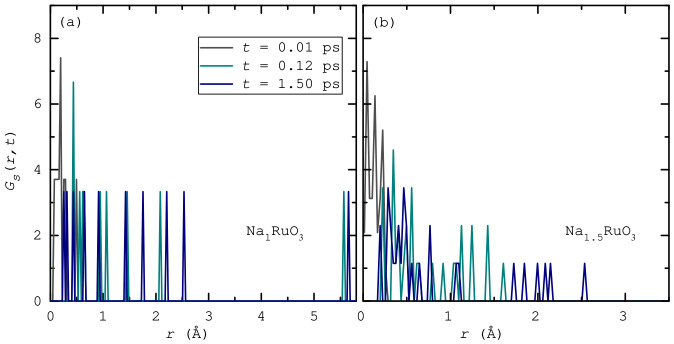
Self van Hove correlation functions $G_{s}\left(r,t\right)$ of Na ions derived from ab initio molecular dynamics simulations histogrammed over 200 bins: (**a**) Na_1_RuO_3_ and (**b**) Na_1.5_RuO_3_.

For Na_1_RuO_3_ (Figure 5a), in the beginning at t=0.01 ps, $G_{s}\left(r,t\right)$ is sharply peaked just above r=0, reflecting pure vibrational motion of Na ions around their equilibrium positions. After a while, at t=0.12 ps, the peak broadens only modestly and does not develop pronounced long-distance tails. By the end of the AIMD run at t=1.5 ps, this description remains almost unchanged. The absence of secondary peaks or extended tails indicates that Na motion is dominated by local rattling within confined coordination environments, with no evidence of site-to-site hopping on the simulated timescale. Consequently, Na ions in Na_1_RuO_3_ experience relatively strong confinement, consistent with the intrinsic cation-vacancy topology of the ilmenite structure, which allows large-amplitude but nonetheless strongly localised vibrations.

In contrast, the $G_{s}\left(r,t\right)$ functions for Na_1.5_RuO_3_ (Figure 5b) exhibit broader displacement distributions that evolve more markedly with time. While at the beginning (t=0.01 ps), a sharp localised peak indicates vibrational motion, as in Na_1_RuO_3_, the increased weight at larger *r* values indicates that Na ions explore larger local volumes, consistent with weaker confinement. In this case, although a fully developed diffusive tail is not observed, the progressive widening of $G_{s}\left(r,t\right)$ suggests incipient hopping or intermittent migration into nearby voids. One possible explanation might be the Na-excess environment in Na_1.5_RuO_3_, where additional Na occupies transition-metal layers and creates Na–O–Na motifs, leads to a softer potential landscape that permits larger-amplitude Na motion and enhanced dynamical heterogeneity.

**Figure 6:**
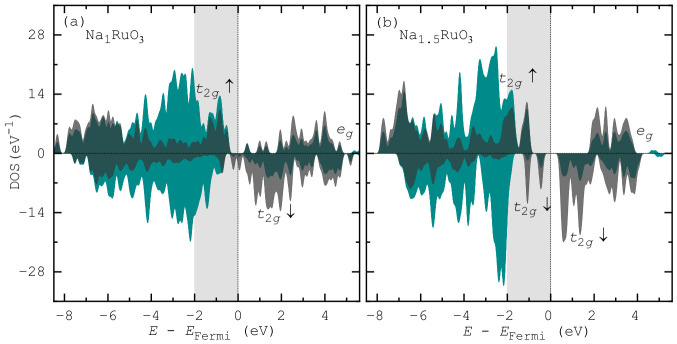
The Ru 4d and O 2p partial density of states (PDOS) represented by grey and green shades, respectively: (**a**) Na_1_RuO_3_ and (**b**) Na_1.5_RuO_3_. The crystal field splittings of the Ru 4d states are marked. The 2 eV window below the Fermi level (shaded in light grey) is co-occupied by both Ru and O states.

Finally, we turn our attention to the electronic structure of these compounds. According to Figure 6, the spin-resolved partial density of states (PDOS) of both Na_1_RuO_3_ and Na_1.5_RuO_3_ show broadly similar qualitative electronic features, with Ru 4d states dominating near the Fermi level and strong hybridisation with O 2p states in the ∼2 eV window below it (which is most important for redox), hinting at a similar covalency in both compounds. In each case, the Ru 4d manifold exhibits clear crystal-field splitting, driven by the octahedral environment, into lower $t_{2g}$ and higher $e_{g}$ states, with comparable overall bandwidths. However, Na_1.5_RuO_3_ shows a noticeable redistribution of states near the Fermi level, with increased occupation and slight broadening of the Ru $t_{2g}$ bands, as well as enhanced spin asymmetry compared to Na_1_RuO_3_. This suggests that the higher Na content modifies the electronic filling and local environment, leading to subtle changes in the electronic structure while preserving the fundamental Ru–O hybridised framework.

Integrating PDOS near the Fermi level quantifies electronic lability by revealing the electronic population of each species most accessible to redox. A higher integrated population in this energy window indicates greater participation of a specific element, thereby pinpointing its relative contribution to charge compensation during (de)sodiation [4]. Here, in Na_1_RuO_3_, the PDOS integration within the 2 eV, as shaded in Figure 6a, yields O and Ru populations of 0.689 *e* and 0.909 *e* per atom, respectively. Consequently, O 2p states are expected to bear 43% of the redox capacity, which is 1.597 *e* in total. In contrast, for Na_1.5_RuO_3_, the same analysis gives O and Ru populations of 0.321 *e* and 0.998 *e* per atom, respectively, corresponding to an oxygen contribution of about 24%. This reduction highlights a shift toward more Ru-driven redox behaviour as the Na content increases.

In Na_x_RuO_3_, earlier experiments showed that anionic redox is reversible [11]. However, this is not generally the case. Irreversible O redox results in hazardous O release [28]. In general, anionic redox is not reversible or stable in many Na-based transition metal (TM) oxides (Na_x_TM_y_O_z_). Strategies to prevent irreversible oxygen redox in such compounds focus on lattice stabilisation and electronic modulation. Doping with elements such as Ti, Ru, Cu, Mg, and Li in the TMO_6_ suppresses Jahn-Teller distortions and TM charge disproportionation, thereby mitigating structural degradation [29,30]. Constructing ordered superlattices with ribbon or honeycomb symmetries, or optimising Na distribution via Zn/Ti dual doping, promotes stable solid-solution reactions and inhibits irreversible transition-metal migration [11,31,32]. These modifications, alongside surface dielectric coatings and reduced interlayer O–O repulsion, stabilise O 2p states, thereby suppressing detrimental oxygen loss and gas evolution [30,32,33]. Finally, fostering specific local bonding environments (e.g., Li–O–Na or Cu–O) reinforces structural integrity, ensuring long-term reversible anionic redox without irreversible phase transitions [31,33,34,35].

## 4. Conclusions

In conclusion, this research, using ab initio molecular dynamics to study sodium ruthenates, demonstrates several key insights. Sodium ions are significantly more mobile than the Ru–O framework, exhibiting notably higher (up to threefold) mean-squared displacement (MSD) values in both Na_1_RuO_3_ and Na_1.5_RuO_3_. Although Na dynamics are primarily characterised by confined rattling within coordination environments with no evidence of long-range site-to-site hopping at room temperature, the Na_1.5_RuO_3_ phase facilitates larger local volume exploration and incipient migration into nearby voids due to a softer potential landscape. Analysis of the radial distribution functions demonstrates that the excess Na in Na_1.5_RuO_3_ results in more pronounced Na–Na interactions and tighter, more ordered Na–O coordination. Despite these differences, the underlying Ru–O framework remains well preserved in both compounds, with consistent bond distances. Finally, electronic structure analysis reveals a clear shift in redox mechanism: the oxygen contribution to redox capacity drops from approximately 43% in Na_1_RuO_3_ to 24% in Na_1.5_RuO_3_, highlighting a decrease in O-driven redox activity with increasing sodium content.

## Data Availability

The original contributions presented in this study are included in the article. Further inquiries can be directed to the author.
